# Efficacy of tranexamic acid application in gynecology and obstetrics procedures: a umbrella review of systematic reviews of randomized trials

**DOI:** 10.61622/rbgo/2025rbgo18

**Published:** 2025-04-30

**Authors:** Nicole Cristina Lottermann, Nathalia Luiza Andreazza, Matheus de Araújo Moura Cavalcante, Laura Andrade Fernandez, Carla Vitola Gonçalvez, Linjie Zhang

**Affiliations:** 1 Universidade Federal do Rio Grande Rio Grande RS Brazil Universidade Federal do Rio Grande, Rio Grande, RS, Brazil.

**Keywords:** Tranexamic acid, Cesarean section, Uterine myomectomy, Hemorrhage, Efficacy, Obstetric surgical procedures, Gynecologic surgical procedures, Hematocrit, Blood transfusion, Hysterectomy, Uterine cervical dysplasia

## Abstract

**Objective:**

This umbrella review aimed to synthesize evidence from systematic reviews of clinical trials on the efficacy of tranexamic acid in gynecology and obstetrics procedures.

**Methods:**

We searched Medline, Embase, SciELO and Cochrane Database of Systematic Reviews on March 11, 2024, using the term "tranexamic acid". Four reviewers independently select studies and extract data. We assessed the quality of systematic review and the quality of evidence, using AMSTAR 2 and GRADE tools, respectively.

**Results:**

Of 651 systematic reviews identified, 16 reviews with 96663 patients were included. The surgical procedures were cesarean section, myomectomy, hysterectomy, and cervical intraepithelial neoplasia surgery. All reviews showed a statistically significant and clinically relevant reduction in intraoperative and post-procedure blood loss, associated with intravenous or topical use of tranexamic acid. Tranexamic acid resulted in a significant reduction in the need for blood transfusions and a less pronounced drop in postoperative hematocrit and hemoglobin levels in cesarean section. Several reviews addressed the same question, but the number of included trials varied substantially, which might indicate flaws in search and selection of studies of these reviews. The quality of systematic reviews was low or critically low, and the quality of evidence was moderate.

**Conclusions:**

This umbrella review shows that tranexamic acid can reduce blood loss and hemorrhage in gynecology and obstetrics procedures. High quality systematic reviews are still needed.

## Introduction

Tranexamic acid (TXA) is a synthetic derivative of the amino acid lysine that exerts its antifibrinolytic effect through a reversible blockade of lysine binding sites on plasminogen.^(1)^ The benefits of TXA in reducing bleeding complications are well-documented by randomized trials in both medical and surgical scenarios.^(2-4)^

TXA has been receiving increasing attention in gynecology and obstetrics due to its pharmacological property, low cost, accessibility, and good safety profile.^(5)^ TXA is recommended by the World Health Organization (WHO) for the treatment of postpartum hemorrhage_._^(6)^ However, current perioperative guidelines in gynecologic surgery do not include recommendations for or against the use of TXA as a preoperative or intraoperative adjunct.^(7)^ High-quality evidence is needed to support evidence-based recommendations on the use of TXA in gynecology and obstetrics.

In the last decade, several systematic reviews of clinical trials have been carried out to evaluate the effects of TXA in gynecology and obstetrics procedures, especially cesarean section.^(8,9)^This umbrella review aimed to synthesize the evidence from these systematic reviews.

## Methods

We conducted and wrote this umbrella review following the Joanna Briggs Institute´s guidelines and PRISMA (Preferred Reporting Items for Systematic Reviews and Meta-Analyses) guidelines.^(10,11)^

### Inclusion and exclusion criteria

Systematic reviews of clinical trials with or without meta-analysis that met the following PICO criteria were included: 1) Participants: adults undergoing gynecology and obstetrics procedures; 2) Interventions: systemic or topical application of TXA 3) Comparisons: placebo, other drugs or no intervention, and 4) Outcomes: primary - blood loss and post-procedure hemorrhage; secondary - blood transfusion, additional use of uterotonic agents, hemoglobin and hematocrit levels. Narrative and systematic reviews or meta-analyses that address other surgical or medical procedures were excluded.

### Sources and search strategy

We searched Medline, Embase, SciELO and Cochrane Database of Systematic on March 11, 2024, using the term "tranexamic acid". In order to identify as many systematic reviews as possible, we used the search strategy with a single term.

### Study selection

Four authors (NCC, NLA, MAMC, LF) independently assessed the titles and abstracts of all citations identified by the searches, using Rayyan platform.^(12)^ We obtained the full articles when they met the inclusion criteria or there were insufficient data in the title and abstract for assessment of eligibility. The definitive inclusion of studies was made after reading the full-text articles. Any disagreement between the four reviewers regarding study inclusion was solved by the senior reviewer (LZ).

### Data extraction

Two reviewers (NCC, NLA) independently extracted the data of each selected study. A form was designed to extract five data categories: 1) Study identification (first author's name, year of publication, country of origin), study aim and funding; 2) Type of participants and procedures; 3) Routes and doses of TXA, and comparisons; 4) Type of outcomes; 5) Results: pooled mean difference (MD) and 95% confidence interval (95% CI) for continuous outcomes; pooled risk ratio (RR) or odds ratio (OR) and 95% CI for dichotomic outcomes.

### Assessment of study quality and quality of evidence

Two reviewers (NCC, NLA) independently assessed the methodological quality of each review, using "A MeaSurement Tool to Assess systematic Reviews 2" (AMSTAR 2).^(13)^ The quality of the review was classified as high, moderate, low and critically low. Any disagreement between the two reviewers regarding study quality assessment was solved by the senior reviewer (LZ).

The quality of evidence was assessed using the Grading of Recommendations Assessment Development and Evaluation (GRADE) tool.^(14)^ We used the quality of evidence reported by the review if GRADE tool was applied for assessment. Otherwise, Two reviewers (MAMC, LF) independently assessed the quality of evidence, based on the data extracted from the review or included trials. Any disagreement between the two reviewers regarding evidence quality assessment was solved by the senior reviewer (LZ).

## Results

**Figure 1:**
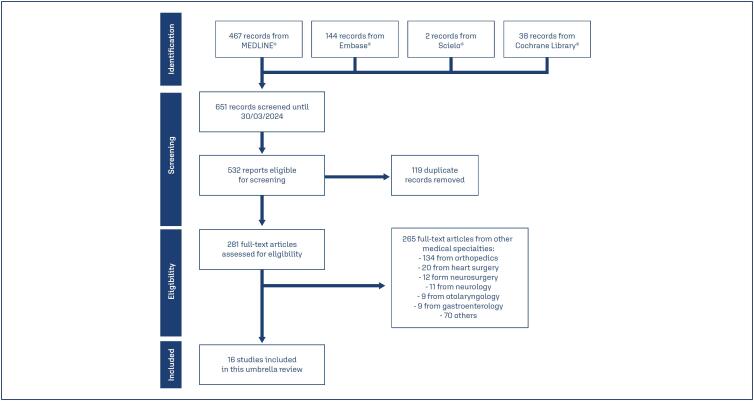
PRISMA flowchart for study identification and selection.

The search strategy identified 532 unique systematic reviews. After screening the titles and abstracts, we retrieved 298 potentially relevant full-text articles for further evaluation. 16 reviews^(15-30)^ involving 96,663 patients were included in this umbrella review (Figure 1). All selected systematic reviews included only randomized trials and were published between the years 1999-2023, three in China,^(28-30)^ two in the USA,^(25, 26)^ two in Italy,^(22,23)^ two in Saudi Arabia,^(17,19)^ one in Canada,^(16)^ one in South Africa,^(24)^ one in the United Kingdom,^(15)^ one in Denmark,^(27)^ one in Greece,^(20)^ one in Brazil^(18)^ and one in Pakistan.^(21)^ Most studies reported no conflicts of interest and received public funding.

### Characteristics of included studies

The main characteristics of studies included in this umbrella review are summarized in chart 1. Regarding the type of procedures, ten reviews evaluated the use of TXA in cesarean section,^(18,20-23,25,26,28-,30)^ three in myomectomies,^(16,19,24)^ one in cervical intraepithelial neoplasia,^(15)^ one in hysterectomy,^(17)^ and one in both cesarean section and myomectomies.^(27)^

**Chart 1:** Main characteristics of the included systematic reviews

Systematic review	Study aim	PICO				Type of surgery	Drug administration	Number os studies and patients
		Population (P)	Intervention (I)	Comparison (C)	Outcome (O)			
Abu-Zaid et al. 2022)^(17)^	Conduct a meta- analysis of RCTs that evaluated the clinical applications of prophylactic TXA	Women undergoing hysterectomy	Prophylactic TXA	None or placebo	Duration of surgery, length of hospital stay, intraoperative blood loss, need for post-procedure transfusion, hemoglobin levels, need for intra- operative topical homeostatic agents, frequency nausea and vomiting, diarrhea	Hysterectomy	IV or topical	5 (911)
Assis et al. (2023)^(18)^	To evaluate the effectiveness of TXA administration in the reduction of intrapartum blood loss and prevention of postpartum hemorrhage	Women undergoing cesarean section	Prophylactic TXA	Placebo	Primary: Peripartum blood loss Secondary: Hemoglobin levels, need for uterotonic agents and need for use of blood derivatives	Cesarean section	IV	16 (6701)
Baradwan et al. (2022)^(19)^	To conduct a systematic review and meta-analysis of randomized controlled trials on the clinical efficacy and safety of TXA in myomectomy	Women undergoing myomectomy	Prophylactic TXA	None or saline solution	Intraoperative, postoperative and total blood loss, hemoglobin and hematocrit level, hospital stay, average operation time, need for blood transfusion, nausea and thromboembolic effects	Myomectomy	IV or topical	7 (571)
Bellos and Pergialiotis (2022)^(20)^	Evaluate the efficacy and safety of TXA in cesarean sections	Women undergoing cesarean section	Prophylactic TXA	Placebo	Total blood loss, severe hemorrhage (>1000 mL), hemoglobin level, need for transfusion, need for additional uterotonics, thromboembolic events	Cesarean section	IV	36 (10659)
Cheema et al. (2023)^(21)^	To evaluate the efficacy and safety of TXA in preventing postpartum hemorrhage in low- and high-risk cesarean sections	Women undergoing cesarean section	Prophylactic TXA	Placebo	Primary: Total blood loss (>1000 mL), need for transfusion Secondary: >400 mL or 500 mL, reduced hemoglobin levels, need for uterotonic agents, non- thromboembolic adverse events, thromboembolic events, maternal and neonatal morbidity, and maternal and neonatal mortality	Cesarean section	IV	50 (23601)
Ferrari et al. (2022)^22^	Summarize available data and evidence on the role of TXA in the prevention and treatment of PPH after vaginal birth and cesarean section	Pregnant women with single-term pregnancies	Prophylactic TXA	Regular clinical practice or placebo	Primary: Blood loss, need for transfusion Secondary: Hysterectomy, maternal death, thromboembolic events.	Cesarean section	IV	22 (33302)
Franchini et al. (2018)^(23)^	To evaluate the safety and efficacy of TXA in reducing preoperative blood loss in cesarean sections.	Pregnant women with single-term pregnancies	Prophylactic TXA	None or placebo	Primary: Postpartum hemorrhage (blood loss > 400mL) and severe postpartum hemorrhage (blood loss > 100mL) Secondary: Need for blood transfusion, average volume of blood loss and occurrence of thromboembolic events	Cesarean section	IV	18 (4557)
Fusca et al. (2019)^(16)^	To evaluate the effectiveness of TXA in reducing perioperative blood loss in myomectomies.	Women of childbearing age undergoing myomectomy	Prophylactic TXA	None or placebo	Perioperative blood loss, need for blood transfusion	Myomectomy	IV or oral	7 (313)
Kongnyuy et al. (2014)^(24)^	To evaluate the efficacy, safety, tolerability, and costs of interventions to reduce intraoperative blood loss in myomectomy.	Premenopausal women undergoing myomectomy	Prophylactic TXA	None or placebo	Primary: Blood loss and need for blood transfusion Secondary: Operational difficulties, postoperative evaluation, safety and tolerability	Myomectomy	IV	12 (674)
Martin-Hirsch et al. (2010)^(15)^	To evaluate the effect of interventions in reducing blood loss in cervical intraepithelial neoplasia surgery	Women undergoing surgical treatment for cervical intraepithelial neoplasia	Prophylactic TXA Vasopressin, hemostatic sutures, Monsel's solution	Unspecified	Short-Term Complications: Objective and subjective perioperative bleeding, primary and secondary hemorrhage Long-term complications: Amenorrhea, dysmenorrhea, satisfactory colposcopy and cervical stenosis	Cervical intraepithelial neoplasia	IV	7 (1179)
Simonazzi et al. (2016)^(25)^	To evaluate the effectiveness of TXA in reducing blood loss when administered before cesarean section.	Women undergoing cesarean section	Prophylactic TXA	None or placebo	Primary: Postpartum blood loss Secondary: incidence of postpartum hemorrhage (blood loss >500 mL), severe postpartum hemorrhage (blood loss >1,000 mL), use of additional medical interventions to control postpartum hemorrhage, thromboembolic events, drop in 24-hour hemoglobin and hematocrit, transfusions during or immediately after cesarean section, severe maternal morbidity and maternal adverse drug reactions	Cesarean section	IV	9 (2365)
Stortroen et al. (2020)^(26)^	To evaluate the effectiveness of prophylactic treatment with TXA in patients at high risk for postpartum hemorrhage.	Women undergoing cesarean section	Prophylactic TXA	Placebo	Primary: total intraoperative blood loss and total postoperative blood loss. Secondary: Need for additional uterotonic agents, change in postoperative hemoglobin concentration and number of patients requiring blood transfusion	Cesarean section	IV	3 (203)
Topsoee et al. (2017)^(27)^	To evaluate the antihemorrhagic effect of prophylactic TXA treatment in major benign uterine surgeries.	Women undergoing elective major benign uterine surgery	Prophylactic TXA	None or placebo	Primary: Operative blood loss (intra-operative or total blood loss) Secondary: Total blood loss >500 mL or >1,000 mL, blood transfusion and thromboembolic events Total, intraoperative and postpartum blood loss, hemoglobin decline, hematocrit decline, incidence of postpartum hemorrhage, incidence of blood loss >1000mL and need for blood transfusion	Cesarean section and myomectomies	IV	16 (2949)
Wang et al. (2015)^(28)^	To evaluate the effect of TXA in reducing intra and postoperative blood loss in cesarean section	Women undergoing cesarean section	Prophylactic TXA	None or placebo	Total, intraoperative and postpartum blood loss, hemoglobin decline, hematocrit decline, incidence of postpartum hemorrhage, incidence of blood loss >1000mL and need for blood transfusion	Cesarean section	IV	11 (2531)
Wang et al. (2019)^(29)^	Evaluate the effectiveness of prophylactic TXA in hemorrhages in cesarean sections	Women undergoing cesarean section	Prophylactic TXA	None or placebo	Perioperative blood loss and postoperative, blood transfusion, massive hemorrhage, additional use of uterotonic agent, total blood loss, Hb change and Hct change	Cesarean section	IV	21 (3852)
Yang et al. (2023)^(30)^	To evaluate the effect of TXA on perioperative blood loss in cesarean sections by systematic review and meta- analysis of published studies	Women undergoing cesarean section	Prophylactic TXA	None or placebo	Blood loss during cesarean section, blood loss 2h after birth, postpartum blood loss (during cesarean section and 2h postpartum), hemoglobin levels	Cesarean section	IV	9 (2295)

The route of administration of TXA was intravenous (IV) in all but three reviews.^(16,17,19)^ These three reviews used both IV and oral, either IV or oral, and either IV or topical TXA. The dose of TXA varied depending on the type of procedure. In the reviews that the surgical procedure was a cesarean section, the dose of TXA was 1g or 10-20mg/kg (maximum 1g) intravenous (IV) bolus before skin incision.^(18,20-23,26,28-30)^ In the reviews that the procedure was myomectomy, most of the trials used a dose of 1g or 10-20mg/kg (maximum 1g) IV bolus before the skin incision, associated with 1mg/kg/h until the end of the procedure or for 6 to 10 hours.^(16,19,24,27)^ In the review that evaluated the use of TXA during hysterectomy, the dose was 10mg/kg IV bolus.^(17)^ In the review that evaluated the use of TXA in surgery for cervical intraepithelial neoplasia, the dose was 1g IV bolus associated with 1g orally for 14 days.^(15)^

## Efficacy of TXA - primary outcomes

### Blood loss

The 16 selected reviews addressed blood loss regardless of the procedure. Of these, nine addressed intraoperative blood loss,^(16,17,19,24,26-30)^ eight addressed postoperative blood loss,^(15,16,19,23,26,28-30)^ and eight addressed total blood loss.^(18-21,27-30)^ All reviews found a statistically significant reduction in blood loss when comparing the intervention with the control (Chart 2). The methods used to quantify blood loss and time points of measurements varied substantially between the reviews.

**Chart 2:** Blood loss (mL), results of the systematic reviews

Systematic review	Number of studies and patients	Type of surgery	MD (IC 95%)	p-value
Intraoperative blood loss				
Abu-Zaid et al. (2022)^(17)^	6 (804)	Hysterectomy	-143.15 (-190.21, -96.09)	<0.001
Baradwan et al. (2022)^(19)^	7 (546)	Myomectomy	-224.34 (-303.06, -145.61)	<0.001
Fusca et al. (2019)^(16)^	2 (166)	Myomectomy	-213.1 (-242.4, -183.7)	<0.05
Kongnyuy et al. (2014)^(24)^	1 (100)	Myomectomy	-243 (-460.02, -25.98)	0.028
Stortroen et al. (2020)^(26)^	2 (182)	Cesarean section	-361.41 (-573.12, -149.69)	0.0008
Topsoee et al. (2017)^(27)^	11 (1919)	Cesarean section	-136 (-189, -83)	<0.00001
	2 (160)	Myomectomy	-251 (-391, -110)	0.0005
Wang et al. (2015)^(28)^	7 (1458)	Cesarean section	-143.36 (-220.38, -66.35)	0.01
Wang et al. (2019)^(29)^	14 (1665)	Cesarean section	-155.23 (-195.64, -114.81)	<0.01
Yang et al. (2023)^(30)^	7 (1332)	Cesarean section	-170.92 (-215.28, -126.55)	<0.00001
Post-procedure blood loss				
Baradwan et al. (2022)^(19)^	4 (372)	Myomectomy	-41.65 (-51.59, -31.71)	p< 0.001
Franchini et al. (2018)^(23)^	18 (3607)	Cesarean section	-155.14 (-192.69, -117.58)	<0.00001
Fusca et al. (2019)^(16)^	2 (232)	Myomectomy	56.3 (-67.8, -44.8)	<0.05
Martin-Hirsch et al. (2010)^(15)^	1 (45)	Cervical Intraepithelial neoplasia	-55.6 (-16.29, -94.91)	0.0056
Simonazzi et al. (2016)^(25)^	8 (2275)	Cesarean section	-160.27 (-224.63, -95.92)	<0.00001
Stortroen et al. (2020)^(26)^	3 (242)	Cesarean section	-177.95 (-296.65, -59.25)	0.003
Wang et al. (2015)^(28)^	7 (1458)	Cesarean section	-38.20 (-59.27, -17.12)	0.01
Wang et al. (2019)^(29)^	12 (1398)	Cesarean section	-26.67 (-32.98, -20.36)	<0.01
Yang et al. (2023)^(30)^	3 (300)	Cesarean section	41.32 (-74.78, -7.87)	0.02
Total blood loss				
Assis et al. (2023)^(18)^	12 (978)	Cesarean section	-170.56 (-218.84, -122.29)	0.000
Baradwan et al. (2022)^(19)^	3 (240)	Myomectomy	-368.12 (-424.17, -312.07)	<0.001
Bellos and Pergialiotis (2022)^(20)^	36 (10659)	Cesarean section	-189.44 (-218.63, -160.25)	NI
Cheema et al. (2023)^(21)^	High risk 6 (376)	Cesarean section	High risk -377.89 (-449.44, -306.33)	High risk <0.00001
	Low risk 41 (11465)		Low risk -179.97 (-203.67, -156.26)	Low risk <0.00001
Topsoee et al. (2017)^(27)^	8 (2115)	Cesarean section	-145 (-196, -96)	<0.00001
Wang et al. (2015)^(28)^	9 (12351)	Cesarean section	-141.61 (-207.09, -76.14)	<0.01
Wang et al. (2019)^(29)^	12 (2901)	Cesarean section	-184.88 (-218.83, -150.94)	<0.01
Yang et al. (2023)^(30)^	2 (800)	Cesarean section	-229.81 (-331.48, -128.14)	<0.00001

MD: mean difference; 95% CI: 95% confidence interval; NI: not informed

### Post-procedure hemorrhage

One review assessed hemorrhage after surgery for intraepithelial neoplasia of the cervix.^(15)^ All other reviews evaluated post-cesarean hemorrhage.^(20-23,25,27-30)^ Six reviews evaluated mild postpartum hemorrhage (>400-500mL),^(15,21,23,25,27,28)^ and nine evaluated severe postpartum hemorrhage (>1000mL).^(15,20-23,25,27-29)^ For mild post-procedure hemorrhage, all but one review^(15)^ showed a significant reduction. All reviews showed a significant reduction in severe post-procedure hemorrhage (Chart 3).

**Table 3:** Post-procedure bleeding, results of systematic reviews

Systematic review	Number of studies and patients	Type of surgery	RR (CI 95%)	p-value
Hemorrhage (>400-500mL)				
Cheema et al. (2023)^(21)^	Low risk 10 (6176)	Cesarean section	0.30 (0.17, 0.53)	0.06
Franchini et al. (2018)^(23)^	5 (786)	Cesarean section	0.40 (0.24, 0.65)	0.0003
Martin-Hirsch et al. (2010)^(15)^	1 (360)	Intraepithelial neoplasia of the cervix	0.23 (0.13, 0.42)	NI
Simonazzi et al. (2016)^(25)^	9 (2365)	Cesarean section	0.21 (0.16, 0.28)	NI
Topsoee et al. (2017)^(27)^	3 (374)	Cesarean section and myomectomy	0.52 (0.35, 0.77)	NI
Wang et al. (2015)^(28)^	4 (1104)	Cesarean section	0.57 (0.37, 0.89)	0.01
Severe hemorrhage (>1000mL)				
Bellos and Pergialiotis (2022)^(20)^	10 (6867)	Cesarean section	0,37* (0.22, 0.60)	NI
Cheema et al. (2023)^(21)^	High risk 3 (308)	Cesarean section	High risk 0.26 (0.17, 0.42)	High risk 0.00001
	Low risk 15(16367)		Low risk 0.64 (0.51, 0.81)	Low risk 0.0002
Ferrari et al. (2022)^22^	15 (4911)	Cesarean section	0.84 (0.75, 0.94)	¼ 0.003
Franchini et al. (2018)^(23)^	5 (1750)	Cesarean section	0.32 (0.12, 0.84)	0.02
Martin-Hirsch et al. (2010)^(15)^	3 (633)	Intraepithelial neoplasia of the cervix	0.23 (0.13, 0.42)	<0.00001
Simonazzi et al. (2016)^(25)^	9 (2365)	Cesarean section	0.42 (0.19, 0.92)	NI
Topsoee et al. (2017)^(27)^	3 (1524)	Cesarean section and myomectomy	0.38 (0.18, 0.81)	NI
Wang et al. (2015)^(28)^	2 (1400)	Cesarean section	0.43 (0.2, 0.92)	0.03
Wang et al. (2019)^(29)^	9 (2358)	Cesarean section	0.39 (0.30, 0.51)	<0.01

Abbreviations: RR: relative risk; 95% CI: 95% confidence interval; NI: not informed

* Outcome was evaluated using the odds ratio parameter.

### Efficacy of TXA - Secondary outcomes

#### Need for blood transfusion

Twelve reviews evaluated this outcome, eight in post-cesarean section,^(20,21,23,25,27-29)^ three in post-myomectomy^(16,19,24)^ and one after hysterectomy.^(17)^ For cesarean section and hysterectomy procedures, all reviews showed a significant reduction in the need for transfusion. Regarding the myomectomy procedure, all but one review^(24)^ reported a significant reduction in the need for transfusion (Supplementary table 1).

#### Additional use of uterotonic agents

Five reviews that assessed this outcome^(20,21,25,26,29)^ reported a significant reduction in additional use of uterotonic agents (Supplementary table 2).

#### Postoperative hemoglobin and hematocrit levels

Eleven reviews evaluated postoperative hemoglobin levels, of which three evaluated after the myomectomy procedure,^(16,19,24)^ seven after the cesarean section^(20,21,25,26,28-30)^ and one after the hysterectomy procedure.^(17)^ In two reviews that addressed myomectomies,^(16,19)^ patients treated with TXA had a higher absolute hemoglobin level after procedures. In the remaining reviews, the difference between TXA and controls was not statistically significant. In all but two reviews,^(25,30)^ higher levels of postoperative hemoglobin were observed in cesarean section (Supplementary table 3).

Four reviews evaluated postoperative hematocrit levels, two in a myomectomy procedure^(19,24)^ and two in a cesarean section.^(25,28)^ The use of TXA was associated with an increased level of hematocrit in the cesarean section (Supplementary table 4, 5 and 6).

## Discussion

This umbrella review provides a systematic synthesis of evidence from systematic reviews of randomized trials on the effects of TXA in gynecology and obstetrics procedures. It also allows us to compare the quality and number of included trials between systematic reviews that addressed the same PICO question.

### Efficacy of tranexamic acid

Most of systematic reviews included in this umbrella review showed benefits of use of tranexamic acid for the primary outcomes, that is, it resulted in a significant reduction in both blood loss and post-procedure hemorrhage in gynecology and obstetrics procedures. Regarding secondary outcomes, such as the need for blood transfusion and hemoglobin and hematocrit levels, tranexamic acid also had beneficial effect in cesarean section. However, the results were not statistically significant for myomectomy and hysterectomy. Use of tranexamic acid also significantly reduced additional use of uterotonic agents in cesarean section. It is important to note that systematic reviews included in this umbrella review addressed different gynecology and obstetrics procedures, such as cesarean section, myomectomy, hysterectomy, and neoplasia surgery. Moreover, they also assessed different variables. Despite the heterogeneity between reviews, most of them showed beneficial effects of tranexamic acid on primary outcomes, but it might be responsible for the variation of the results in some secondary outcomes.

### Quality of reviews and evidence

Many systematic reviews published in consecutive years addressed the same PICO question. However, there was a large discrepancy between these reviews in the number of included trials, which might indicate flaws in search and selection of primary studies of these reviews. This methodological limitation can cause biases in review results and waste of research resources. Furthermore, according to the AMSTAR 2 tool, the majority of systematic reviews are of critically low or low quality (Supplementary table 5). The methodological limitations may raise concerns about applicability of the findings of these reviews in gynecology and obstetrics practice. This occurred mainly because most reviews did not adequately address the critical domain 7 (description of excluded studies and reasons) and 15 (publication bias) of the AMSTAR 2 tool. Lack of information on excluded studies and publication bias might result in bias in the conclusion of the reviews. It highlights the need for improving the quality of future systematic reviews. Regarding the quality of evidence in the reviews, most of them were classified as moderate quality (Supplementary table 6). This classification is mainly due to the inconsistency of the results according to GRADE. Another domain is heterogeneity which was present in most of the reviews.

## Conclusion

Moderate-quality evidence from systematic reviews of randomized trials shows the benefits of tranexamic acid in reducing blood loss and hemorrhage in gynecology and obstetrics procedures. High quality systematic reviews are still needed.

## Supplementary Material

### Table 1S Need for blood transfusion, results of systematic reviews

**Table:**

Systematic review	Number of studies	Type of surgery	RR/OR (IC95%)	Value-p
Abu-Zaid 202217	4 (604)	Hysterectomy	0.31 (0.16, 0.58)	<0.001
Baradawan 202219	6 (504)	Myomectomy	0.53 (0.28, 1.01)	0.05
Bellos 202220	14 (7177)	Cesarean section	OR 0.41 (0.26, 0.65)	NI
Cheema 202321	High risk: 5 (530) Low risk: 24 (19384)	Cesarean section	High risk: 0.28 (0.17, 0.44) Low risk: 0.48 (0.35, 0.68)	0.00001
Franchini 201823	10 (1873)	Cesarean section	0.30 (0.18, 0.49)	0.00001
Fusca 201916	2 (166)	Myomectomy	0.58 (0.33, 1.00)	<0.05
Kongnyuy 201424	1 (100)	Myomectomy	1.71 (0.68, 4.30)	0.25
Simonazzi 201625	9 (2365)	Cesarean section	0.33 (0.19, 0.58)	<0.00001
Topsoe 201727	4 (968)	Cesarean section and myomectomy	0.32 (0.17, 0.59)	<0.00001
Wang 201528	4 (1047)	Cesarean section	0.23 (0.10, 0.57)	<0.01
Wang 201929	11 (1815)	Cesarean section	0.29 (0.18, 0.49)	<0.01

RR: relative risk; OR: odds ratio; 95%CI: 95% confidence interval.

### Table 2S Use of uterotonic agents, results of systematic reviews

**Table:**

Systematic review	Number of studies and patients	Type of surgery	Effect	RR (IC 95%)	Value-p
Bellos 202220	14 (7680)	Cesarean section	OR	0.36 (0.25, 0.52)	0.05
Cheema 202321	High risk 5 (530) Low risk 17 (19054)	Cesarean section	RR	High risk 0.26 (0.19, 0.37) Low risk 0.56 (0.46, 0.69)	High risk <0.00001 Low risk <0.00001
Stortroen 202026	3 (242)	Cesarean section	RR	0.26 (0.16, 0.41)	<0.00001
Simonazzi 201625	9 (2365)	Cesarean section	RR	0.54 (0.36, 0.81)	<0.00001
Wang 201929	7 (1900)	Cesarean section	RR	0.40 (0.30, 0.55)	<0.01

RR: relative risk; OR: odds ratio; 95%CI: 95% confidence interval.

### Table 3S Postoperative hemoglobin levels, result of the systematic reviews

**Table:**

Systematic review	Number of studies and patients	Tipo de cirurgia e N participantes	DM (95%IC)	Valor-p
Abu-Zaid 2022*17	6 (622)	Hysterctomy	0.61 (0.27, 0.95)	<0.001
Baradawan 2022*19	7 (558)	Myomectomy	0.4 (0.11, 0.68)	0.006
Bellos 2022**20	26 (4674)	Cesarian section	8.22% (5.54, 10.90)	NI
Cheema 2023**21	High risk 6 (576) Low risk 32 (21088)	Cesarian section	High risk 1.07 (0.12, 2.02) Low Risk 0.63 (0.53, 0.74)	High risk <0.00001 Low risk <0.00001
Fusca 2019*16	3 (266)	Myomectomy	0.5 (-0.1, 1.1)	>0.05
Kongnyuy 2014*24	1 (100)	Myomectomy	0.21 (-0.36, 0.78)	0.47
Simonazzi 2015**25	9 (2365)	Cesarian section	-0.61 (-1.04, -0.18)	<0.05
Stortroen 2020*26	2 (182)	Cesarian section	0.41 (-0.08, 0.9)	0.1
Wang 2015**28	6 (1427)	Cesarian section	-0.87 (-1.30, -0.45)	<0.0001
Wang 2019**29	3 (1053)	Cesarian section	0.8 (-1.07, -0.53)	<0.01
Yang 2023**30	5 (1703)	Cesarian section	-0.63 (-0.82, -0.44)	p<0.00001

MD: mean difference; 95%CI: 95% confidence interval.

* Absolute hemoglobin level (g/L)

** Percentage drop in hemoglobin level

### Table 4S Postoperative hematocrit levels, result of the systematic reviews

**Table:**

Review	N estudos	Tipo de cirurgia e N participantes	DM (95% IC)	Valor-p
Baradawan 2022*19	3 (306)	Myomectomy	1.28% (0.13, 2.44)	0.03
Kongnyuy 2014*24	1 (100)	Myomectomy	100 (-0.43, 2.43)	0.17
Simonazzi 2015**25	9 (2365)	Cesarean section	-0.66% (-2.32, -1)	<0.05
Wang 2015**28	2 (313)	Cesarean section	-1.82 (-3.42, - 0.23)	0.02

MD: mean difference; 95%CI: 95% confidence interval; NI: not informed

* Absolute hematocrit level

** Percentage drop in hematocrit level

### Table 5S AMSTAR assessment of the quality level of the included systematic reviews

**Table:**

Systematic review	Level of quality	Criteria not covered
Abu-Zaid 202217	Low	2, 10
Assis 202318	Moderate	10
Baradawan 202219	Very low	2, 10, 15*
Bellos 202220	Moderate	10
Cheema 202321	Moderate	10
Ferrari 202222	Very low	2, 10, 11*, 12*, 15*
Franchini 201823	Very low	7*, 10, 15*
Fusca 201916	Low	10,12,15*
Martin 201015	Very low	2*, 7*,10, 11*,12,13*,15*
Kongnyuy 201124	Very low	7,12,13*,16
Simonazzi 201625	Very low	7*,10,12
Stortroen 202026	Low	10,15*
Topsoe 201627	Very low	7*,10, 15*
Wang 201528	Low	7*,10
Wang 201929	Low	10, 15*
Yang 202330	Very low	10, 15*

* Critical criteria

### Table 6S GRADE assessment of the quality level of evidence of the included reviews

**Table:**

Review	Risk of bias	Imprecision	Inconsistency	Indirectness	Publication bias	Evidence quality
Abu-Zaid 202217	ok	↓	ok	↓	ok	Low
Assis 2023*18	-	-	-	-	-	-
Baradawan 2022*19	-	-	-	-	-	-
Bellos 2022*20	-	-	-	-	-	-
Cheema 2023*21	-	-	-	-	-	-
Ferrari 202222	↓	↓	ok	↓	ok	Very low
Franchini 2018*23	-	-	-	-	-	-
Fusca 201916	ok	↓	ok	ok	ok	Moderate
Kongnyuy2014 24	ok	↓	ok	ok	ok	Moderate
Martin-Hirsch 201015	ok	ok	ok	↓	ok	Moderate
Simonazzi 201625	ok	↓	ok	ok	ok	Moderate
Stortroen 2020*26	-	-	-	-	-	-
Topsoee201727	↓	↓	ok	ok	ok	Low
Wang 201528	ok	↓	ok	ok	ok	Moderate
Wang 2019*29	-	-	-	-	-	-
Yang 202330	ok	↓	ok	ok	ok	Moderate

* Studies that presented their own GRADE
